# An Unusual Case of Severe Aortic Stenosis and Triple-Vessel Coronary Artery Disease in a Patient Presenting With Intermittent Chest Pain

**DOI:** 10.7759/cureus.38705

**Published:** 2023-05-08

**Authors:** Oluwaremilekun Tolu-Akinnawo, Oluwabamise R Akinnawo, Joseph A Akamah

**Affiliations:** 1 Internal Medicine, Meharry Medical College, Nashville, USA; 2 Emergency Medicine, Piedmont Macon North Hospital, Macon, USA; 3 Cardiology, Nashville General Hospital, Nashville, USA; 4 Cardiology, Meharry Medical College, Nashville, USA

**Keywords:** young adult, familiar hyperlipidemia, critical aortic stenosis, three vessel disease (3vd), intermittent chest pain

## Abstract

Although relatively uncommon, premature coronary artery disease (CAD) is a significant cause of mortality and morbidity. A key risk factor for this condition is the presence of familial hyperlipidemia (FH), which is a genetic disorder of impaired cholesterol metabolism. While aortic stenosis is relatively common in the older population, it is rare in young adults, and its presence should raise concern for a bicuspid valve. We present the case of a 37-year-old male patient with intermittent chest pain and physical examination findings of multiple-site xanthomas and echocardiography/angiography findings of severe aortic stenosis and severe three-vessel CAD, respectively. FH was noted as the most critical risk factor in this patient. He was subsequently managed with surgical aortic valve replacement (SAVR) and simultaneous coronary bypass graft surgery with interval symptom improvement. Cholesterol-lowering agents - high-intensity statins/ezetimibe - were started to control cholesterol levels. Guideline-directed medical therapy for CAD/aortic valve replacement (AVR) with beta-blockers, angiotensin-converting enzyme inhibitors, antiplatelet therapy, and warfarin therapy was also employed.

This report highlights a case of acute coronary syndrome (ACS) in a young adult and how early detection and treatment of risk factors can lead to a good prognosis.

## Introduction

Aortic valve stenosis (AS) is the most common form of valvular heart disease in the older population and frequently occurs alongside coronary artery disease (CAD) in this population [1,2]. The risk factors are similar in these two-disease pathologies; therefore, it is not surprising that significant CAD is often present in patients with severe AS [3]. However, AS and CAD remain rare in the younger population, and their presence in this population should trigger additional evaluation. Alongside AS, the prevalence of CAD increases with age, with >50% of patients with AS being above 70 years and >65% of patients over 80 years [4]. Familial hyperlipidemia (FH) is a known risk factor for premature CAD, responsible for an estimated 20% of premature CAD cases [5]. A recent study revealed FH-causative mutations in 3/66 (4.5%) patients screened for premature CAD and a 15-fold increased prevalence of FH mutations in premature CAD compared to the general population [5]. A significant risk factor for severe aortic stenosis in a young patient is a bicuspid valve, which was also suspected in the patient discussed in this report.

## Case presentation

The patient was a 37-year-old Hispanic male with a past medical history of FH and hypertension (HTN) previously not on any medication and was referred for intermittent chest pain after being initially evaluated at an outside facility. The patient reported chest pain that was left-sided, intermittent, with each episode lasting five minutes; it was non-radiating, got worse with exercise, often limiting physical activities, and got better with rest. He also reported associated dyspnea on exertion; however, he denied orthopnea, paroxysmal nocturnal dyspnea (PND), leg swelling, or weight gain. The patient denied diaphoresis, nausea or vomiting, palpitations, or syncopal episodes associated with any of the above symptoms. He reported associated hyperlipidemia in the family; however, there was no history of myocardial infarction. He denied alcohol intake, smoking, or the use of any illicit substances.

Evaluation at the outside facility before referral had been significant for an abnormal lipid panel, requiring high-intensity atorvastatin at a dose of 80 mg daily [repeat low-density lipoprotein was still elevated at 187 mg/dL (reference range: 0-137 mg/dL)]. He also had undergone a 2D echocardiogram (2D-ECHO), which revealed an aortic valve area (AVA) of 1 cm^2^ (reference range: 2.5-4.5 cm^2^), a mean gradient of 24 mmHg (reference value: <5 mmHg), peak velocity of 3.4 m/sec (reference value: <2.5 m/sec), and a left ventricular ejection fraction (LVEF) of 55-65%. Official imaging report from the outside facility was unavailable. Due to his persistent symptoms and discordant echocardiography findings, the patient was referred for a left heart catheterization (LHC) to evaluate the measured pressure gradient and exclude coronary disease.

**Figure 1:**
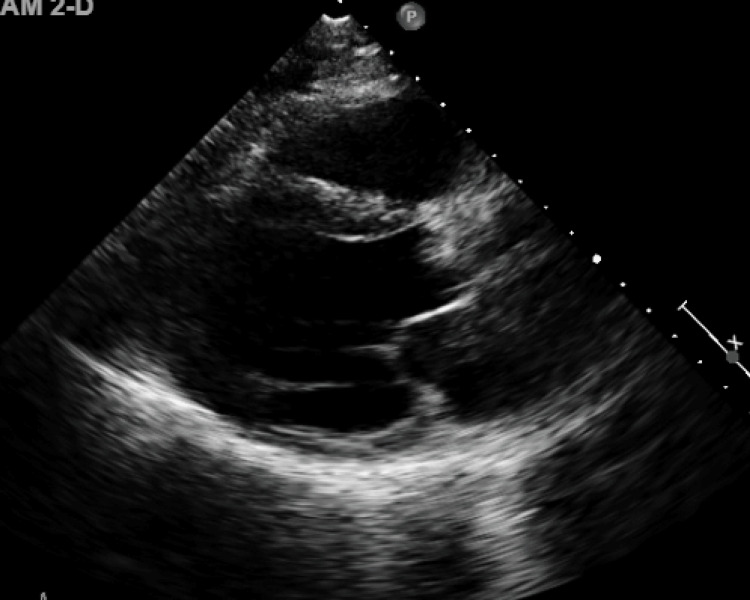
Repeat 2D ECHO revealed LVEF of 40-45% and severe aortic stenosis 2D ECHO: two-dimensional transthoracic echocardiograph; LVEF: left ventricular ejection fraction

At the initial visit, the patient's vital signs were significant for elevated blood pressure (BP) of 155/69 mmHg but otherwise unremarkable. Physical examination was notable for S1, S2, S4, and 4/5 systolic murmur that radiated to the carotid, and multiple-site xanthomas ranging from 1 cm in diameter on the dorsal aspects of his hands to 5 cm on his elbows. Repeat 2D ECHO (Figure 1) was done, which was significant for LVEF of 40-45%, akinesis in basal to the mid inferolateral wall and adjacent basal inferior and anterolateral wall segments, and hypokinetic in other segments, consistent with ischemic heart disease in the distribution of the left circumflex (LCx), and/or right coronary artery (RCA). Markedly thickened and calcified aortic valve with restricted opening, peak aortic valve velocity of 3.82 m/sec (reference value: <2.5 m/sec), a mean gradient of 35 mmHg (reference value: <5 mmHg), AVA of 1.1 cm^2^ (reference range: 2.5-4.5 cm^2^), and mild to moderate aortic valve regurgitation were observed. We were unable to assess if it was trileaflet or bicuspid. There was also mild functional mitral regurgitation.

**Figure 2:**
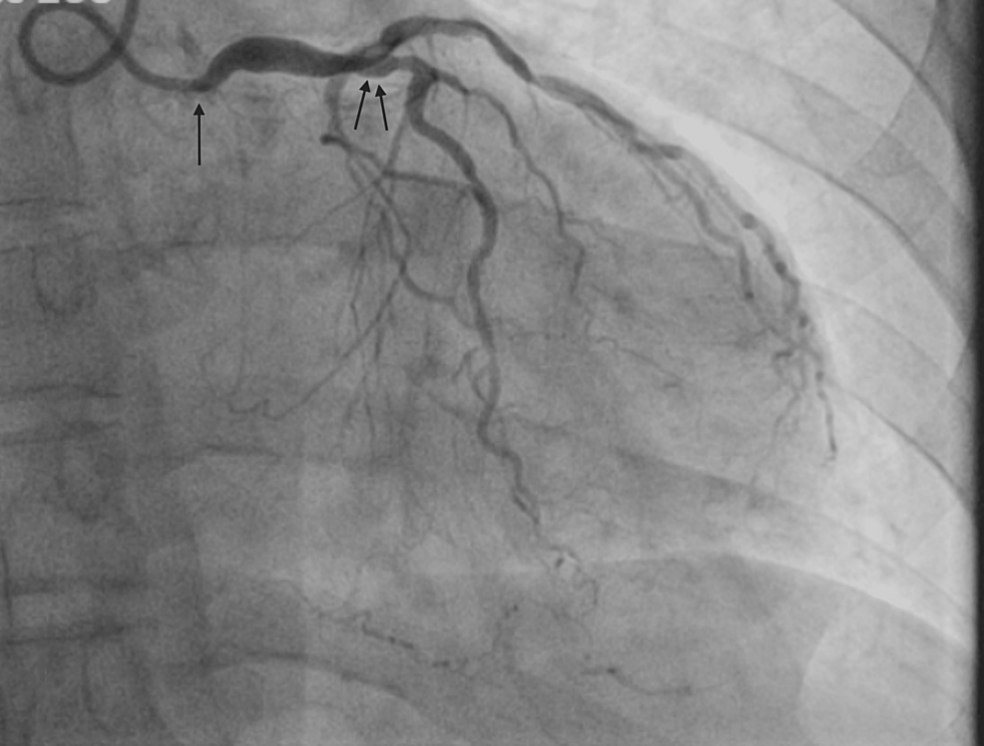
Left heart catheterization revealed severe left main coronary artery stenosis

The patient subsequently underwent an LHC, revealing a peak-to-peak gradient across the aortic valve of about 20 mmHg (reference value: <5 mmHg); left ventricular end-diastolic pressure was severely elevated at 30 mmHg (reference value: 8-12 mmHg). Coronary angiography was significant for a right dominant system with severe left main stenosis and three-vessel CAD. A limited coronary angiography was performed as the patient developed marked ST depression with any engagement of the left main. Figure 2 shows left-sided coronary artery stenosis.

**Figure 3:**
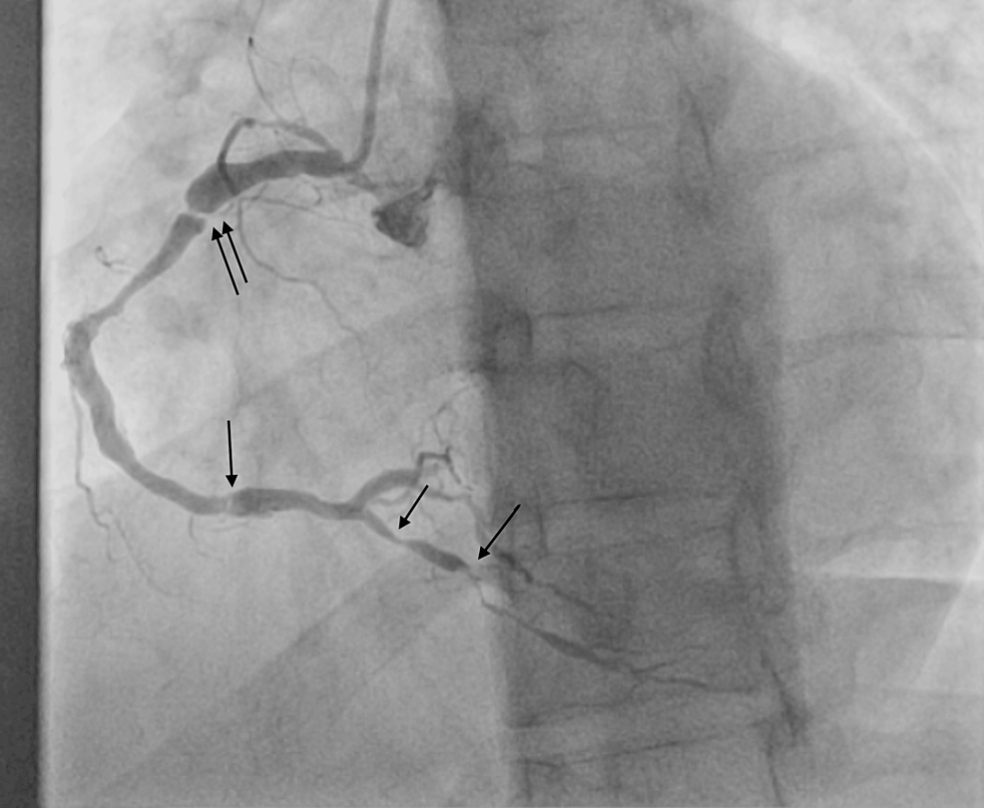
Cardiac catheterization revealed significant right coronary artery stenosis

Figure 3 shows right-sided coronary artery stenosis.

The patient subsequently had surgical aortic valve replacement (SAVR) and coronary bypass surgery with interval symptom improvement. He was also started on clopidogrel and warfarin therapy. Ezetimibe and omega capsules were added to statins therapy, and beta-blocker/angiotensin-converting enzyme inhibitors were also added due to underlying CAD/HTN. The patient was advised to get his siblings and children screened for hyperlipidemia.

## Discussion

Aortic stenosis in young adults (aged 20-65 years) remains rare, with an estimated incidence between 10 and 75 individuals/100,000 per year [6]. Similarly, the prevalence of CAD in patients undergoing SAVR increases with age [7]. A recent Swedish registry demonstrated simultaneous SAVR and coronary artery bypass graft (CABG) in 7.2% of patients aged <50 years, 30.2% of patients aged 51-60 years, 41.2% of patients aged 61-70 years, and 51.2% of patients aged >71 years [7]. Also, in a study of 388 patients with a mean age of 72 years with aortic valve calcification who underwent coronary angiography, there was a significant association between aortic valve calcification and significant CAD [7]. This indicates that aortic valve calcification could serve as a marker for coronary atherosclerotic disease, which could explain the incidence of aortic calcification in patients with FH, as seen in the case of our patient. This is also supported by a post-mortem study of persons aged >65 years, which showed that 100% of people with aortic valve calcification had calcific deposits in one or more coronary arteries [8]. It is, however, essential to consider the bicuspid aortic valve in younger patients with aortic valve stenosis/regurgitation, as in the case of our patient. Studies have shown an increased incidence of congenital bicuspid valves with decreasing age, and it is noted to be the primary reason for aortic stenosis in young adults [6]. Bicuspid valves have a high calcium burden, which, in conjunction with coronary atherosclerotic disease (FH), as in the case of our patient, could be potentially fatal.

FH is an inherited monogenic autosomal dominant disorder of lipid mechanism characterized by elevated serum low-density lipoprotein-cholesterol (LDL-C), whose prolonged exposure is a significant risk factor for acute coronary syndrome (ACS) [9]. It is characterized by cutaneous and, less often, tendinous xanthomas, as seen in our patient. It is also associated with premature corneal arcing and an increased risk of premature CAD in these patients in a pattern similar to atherosclerosis disease from traditional risk factors [9]. FH is often underdiagnosed, leading to delays in treatment and an increased risk of significant high-grade coronary artery stenosis, as seen in our patient [9]. This is supported by a cross-sectional study in which five patients in their second and third decade of life with underlying FH were noted to have significant high-grade coronary stenosis, with three subsequently undergoing a coronary bypass grafting while the remaining two had percutaneous coronary stenting [9]. This has been supported by another study, in which 49 of 66 patients with angiographic evidence of premature CAD had FH mutation [6]. In the case of our patient, in the absence of traditional risk factors (smoking, diabetes mellitus, obesity, poorly controlled HTN) for CAD, FH was recognized as the potential trigger for severe CAD.

A high index of suspicion is required with regard to CAD in patients with severe aortic stenosis due to similar presentations (angina). Given the poor discriminatory capacity of angina in both disease conditions, coronary angiography is often recommended for symptomatic men older than 35 years, asymptomatic men older than 45 years, pre-menopausal women aged more than 35 years with CAD risk factors, post-menopausal women, patients with two or more coronary risk factors, patients with a history of CAD, and patients undergoing cardiac catheterization to evaluate the severity of the valve disease [10].

It is important to note that the simultaneous presence of CAD in patients increases the procedural risk of SAVR, and coronary vascularization is often recommended during surgery. The American College of Cardiology and the American Heart Association have suggested that in patients with aortic valve replacement (AVR) with significant major coronary artery stenosis (>70%), the treatment should involve coronary bypass grafting [10]. The European Society of Cardiology also recommends complete revascularization in patients with severe AS undergoing SAVR to improve long-term outcomes [11]. Although interventions combining AVR and CABG are associated with higher postoperative mortality than AVR alone, it remains the preferred treatment due to the long-term benefits associated with it [12]. A concomitant AVR and percutaneous coronary intervention (PCI) intervention have also been studied and demonstrated potentially reduced mortality rates and hospitalization duration compared to CABG; however, this was a single-center observational study limited to 30-day follow-up [13]. Transcatheter aortic valve replacement (TAVR) has recently emerged as an alternative for high-risk surgical patients. According to a recent study, TAVR combined with PCI within 12 months showed a similar in-hospital mortality in 59 high-risk patients presenting with both CAD and severe AS when compared with 184 high-risk patients undergoing SAVR and CABG surgery [14]. It is also important to treat underlying risk factors - cholesterol-lowering agents for FH in the case of our patient. Guideline-directed medical therapy post-coronary intervention (beta-blockers, angiotensin-converting enzyme inhibitors/angiotensin receptor blockers) and warfarin/dual antiplatelet therapy in the event of aortic valve replacement are also critical. Genetic testing of first-degree relatives of a patient with FH is equally important to prevent the complications that arise with FH.

## Conclusions

This case highlights a severe case of CAD/ACS in a young patient. The providers need to identify high-risk patients and commence preventive strategies early, as this could provide critical prognostic value. Although FH is rare, early treatment with cholesterol-lowering agents could be potentially lifesaving. Early genetic testing of first-degree relatives of patients with FH is critical. SAVR and CABG remain the mainstay of treatment for patients presenting with concomitant CAD and severe AS. Early identification and intervention remain crucial for survival and favorable long-term outcomes.
